# Assessment of the immune landscapes of advanced ovarian cancer in an optimized in vivo model

**DOI:** 10.1002/ctm2.551

**Published:** 2021-10-12

**Authors:** Simone Pisano, Stefania Lenna, Gareth D. Healey, Fereshteh Izardi, Lucille Meeks, Yajaira S. Jimenez, Oscar S Velazquez, Deyarina Gonzalez, Robert Steven Conlan, Bruna Corradetti

**Affiliations:** ^1^ Department of Nanomedicine Houston Methodist Research Institute Houston Texas; ^2^ Center for NanoHealth Swansea University Medical School Swansea UK; ^3^ Texas A&M Health Science Center College of Medicine Bryan Texas

**Keywords:** ascites, CyTOF, immunotherapy, mass cytometry, model, ovarian cancer, peritoneal cancers

## Abstract

**Background:**

Ovarian cancer (OC) is typically diagnosed late, associated with high rates of metastasis and the onset of ascites during late stage disease. Understanding the tumor microenvironment and how it impacts the efficacy of current treatments, including immunotherapies, needs effective in vivo models that are fully characterized. In particular, understanding the role of immune cells within the tumor and ascitic fluid could provide important insights into why OC fails to respond to immunotherapies. In this work, we comprehensively described the immune cell infiltrates in tumor nodules and the ascitic fluid within an optimized preclinical model of advanced ovarian cancer.

**Methods:**

Green Fluorescent Protein (GFP)‐ID8 OC cells were injected intraperitoneally into C57BL/6 mice and the development of advanced stage OC monitored. Nine weeks after tumor injection, mice were sacrificed and tumor nodules analyzed to identify specific immune infiltrates by immunohistochemistry. Ascites, developed in tumor bearing mice over a 10‐week period, was characterized by mass cytometry (CyTOF) to qualitatively and quantitatively assess the distribution of the immune cell subsets, and their relationship to ascites from ovarian cancer patients.

**Results:**

Tumor nodules in the peritoneal cavity proved to be enriched in T cells, antigen presenting cells and macrophages, demonstrating an active immune environment and cell‐mediated immunity. Assessment of the immune landscape in the ascites showed the predominance of CD8^+^, CD4^+^, B^–^, and memory T cells, among others, and the coexistance of different immune cell types within the same tumor microenvironment.

**Conclusions:**

We performed, for the first time, a multiparametric analysis of the ascitic fluid and specifically identify immune cell populations in the peritoneal cavity of mice with advanced OC. Data obtained highlights the impact of CytOF as a diagnostic tool for this malignancy, with the opportunity to concomitantly identify novel targets, and define personalized therapeutic options.

## Graphical abstract

– An ascites‐bearing, metastatic ovarian cancer model was created in mice
– For the first time, cytometry by time of fly (CyTOF) technology was used to delineate the immune landscape of the ascitic fluid
– Our data suggest that the application of this innovative tool will benefit advanced ovarian cancer diagnosis and treatment.

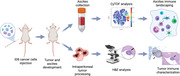

## 1 INTRODUCTION

Ovarian cancer (OC) is the 7th most common cause of death in women worldwide, with over 21 000 new cases expected in the United States in 2020.^1^ Survival rates vary according to the stage of disease, with a 5‐year survival rate of around 30% for advanced cancers,^2^, ^3^ the most common of which is high‐grade serous ovarian carcinoma (HGSOC) accounting for more than 50% of cases. Unfortunately, only 33% of OC cases are identified early, the majority being diagnosed at a later, more advanced stage, and associated with a significantly worse prognosis.^4^

According to the National Comprehensive Cancer Network (NCCN) guidelines, the standard of care therapy for HGSOC involves debulking surgery followed by platinum‐ or taxol‐based chemotherapies. Among other recommended treatments, liposomal doxorubicin is a viable option for both early and advanced‐stage disease.^5^ Targeted therapeutic approaches, recently added to standard clinical practice, provide improved survival rates and include: vascular endothelial growth factor (VEGF)‐A inhibitors,^6^ and poly (ADP‐ribose) polymerase (PARP) inhibitors, which are indicated for patients with a BReast CAncer gene (BRCA1/2) mutation.^5^ OC remains a complex disease to treat, owing to the high chemotherapy‐resistance emergence rate,^7^ and in recent years great emphasis has been placed on the employment of immunotherapies to combat this issue, although currently no clinically approved immunotherapy for HGSOC exists. Modest activity within recurrent OC patients (which included epithelial, fallopian, or primary peritoneal OC) has been reported in the Phase II KEYNOTE‐100 study for the checkpoint inhibitor (CPI) Pembrolizumab.^8^ Additionaly, several Phase III trials are exploring the combination of CPI with PARP or VEGF inhibitors to determine any therapeutic synergies.^9^

Limited immunotherapy efficacy observed to date, however, could be explained by the typically “cold” immune status of OC. Indeed, the advanced OC tumor microenvironment (TME) is characterized by a lack tumor infiltrating lymphocytes (TILs) and failed T‐cell priming due to a combination of poor antigen presentation and an intrinsic insensitivity to T‐cell killing.^10,11^ More specifically, tumor growth is associated with a scarcity (if not total absence) of CD8+ T cells within the TME,^12^ or the inability of dendritic cells (DCs) to effectively present antigen and stimulate a cytotoxic response.^13^ One of the possible mechanisms behind DC inactivation has been provided by Cubillos‐Ruiz et al.^14^ The authors demonstrated that the reduced capability of DC to support an anticancer immune response is associated to the transient, yet abnormal lipid accumulation in the endoplasmic reticulum, which obstructs their normal antigen‐presenting capacity.^14^ Another factor proposed to play a role in ovarian cancer progression at advanced stages and resistance to immunotherapy is the presence of transforming growth factor‐β (TGF‐β). Specifically, TGF‐β is a potent immunosuppressor within the tumor environment being involved in several tumor‐associated processes, including the increase of the epithelial to mesenchymal transition, the promotion of angiogenesis and immune suppression. The enhanced secretion of TGF‐β within the tumor microenvironment is associated to the recruitment of regulatory T cells via expression of FoxP3, which ultimately results in diminished cytotoxic T‐lymphocytes and in a reduced presence of DCs.^15^, ^16^ There is, however, a paucity of evidence on the specific roles of immune cell populations within the OC TME. Hence, a more comprehensive understanding of the immune cell landscape would provide an important platform for the development of more efficacious immunotherapeutic strategies.

The accumulation of fluid within the peritoneal cavity (ascites), which contains a variety of soluble and cellular components, is characteristic of advanced stage OC. Indeed, more than one third of OC patients present with ascites at diagnosis, which has been correlated with its spread within the peritoneal cavity and poor patient prognosis.^17^ The accumulation of ascites occurs as a consequence of unbalanced drainage of the peritoneal cavity, due to obstruction of the lymphatic system by cancer cells,^17^ or by increased leakage of fluid from the microvessels lining the peritoneum.^18^ Ascites build‐up also contributes to malignant progression by facilitating multifocal cancer cell dissemination on the peritoneal surface.^19^ The presence of an intraperitoneal ascitic current, which acts as a means of transport of OC spheroids, further facilitates peritoneal, lymphatic, and hematogenous metastasis,^20^ a phenomenon that falls within the multistep process of metastatic dissemination. Soluble and cellular components within the ascitic fluid have also been shown to influence metastatic behavior.^17^ Soluble components, including growth factors, cytokines, chemokines, and extracellular matrix pieces, inhibit T helper cell proliferation^21^ and DC maturation^22^ mediated by IL‐10. Cellular components, such as resident tumor cells or tumor‐associated fibroblasts, or nonresident immune cells, on the other hand, have a wide ranging impact on the TME. The presence, functionality, and effect of specific, singularly taken immune cell populations within the ascitic fluid has been widely described, unraveling the association between the presence of tumor‐infiltrating CD8+ T cells and the prolonged disease‐free survival,^23^ or unmasking the role of T regulatory cells in creating an immunosuppressive environment.^24^ As such, ascites represents a potentially very informative source of information regarding the effect of immune cells on metastatic disease progression. Moreover, its presence in over 30% of patients at diagnosis renders it an important issue to tackle and explore. Hence, a complete profiling of the ascites immune content would prove useful if done on patients in a tailored fashion. However, fundamental research on the biological interactions of the components of advanced OC ascites requires reliable in vivo models.

In this study, we optimize the development of an advanced OC model in immunocompetent mice to fill the gap in the understanding of the immune landscape within the peritoneal cavity. For the first time, we apply mass cytometry to comprehensively describe the immunological TME within the ascites and to provide insights about the effectiveness of the selected preclinical model in reproducing the human tumor immunomicroenvironment. Finally, we propose mass cytometry as an accurate strategy for the development of personalized strategies against advanced OC and all cancers metastasizing within the peritoneal cavity.

## 2 METHODS

### 2.1 Cell line

The ID8 cell line, originated from mouse ovarian surface epithelial cells (MOSEC), was purchased from Merck‐Millipore. Cells were cultured in High Glucose Dulbecco's Modified Eagle medium (HG‐DMEM) (Sigma) supplemented with 10% fetal bovine serum (FBS, ThermoFisher), 5 μg/mL insulin, 5 μg/mL transferrin and 5 ng/mL sodium selenite (1× ITS, Sigma) and 1× Penicillin‐Streptomycin Solution (Sigma). Culture conditions were 37°C in a humidified 5% CO_2_ atmosphere.

### 2.2 Lentivirus transduction, lentiviral infection of ID8 cells with the luciferase vector and cell line selection

The ID8‐Luc/GFP cell line was generated by transduction with Lentivirus particles containing the CMV promoter for the expression of humanized firefly luciferase (hLUC) and the SV40 promoter for the expression of GFP protein according to manufacturer's protocol (GeneCopoeia). Briefly, ID8 cells were plated at 2 × 10^4^ cells per well (12‐well plate, Corning) and incubated overnight at 37°C in a humidified 5% CO_2_ atmosphere. Cells were then infected with 10 MOI of Lenti‐PAC™ plasmid mix (GeneCopoeia Inc.) in the presence of 8 μg/mL polybrene (Sigma). After overnight incubation at 37°C/5% CO_2_, the viral supernatant was discarded, and cells were washed with 1× PBS (ThermoFisher) prior to the addition of warmed HG‐DMEM media. Three days after infection, cells with high levels of GFP expression were selected by Cell Sorter NIR Aria II (BD Bioscience) and expanded for a week in HG‐DMEM media in presence of 1 μg/mL puromycin (Invitrogen) to further select transfected cells and generate a stable cell line.

### 2.3 In vivo propagation of ID8‐GFP tumors

Female C57BL/6 (5‐6 weeks old) were purchased from the Charles Rivers laboratories. All animal studies were carried out in accordance with guidelines determined by the Animal Welfare Act and the Guide for the Care and Use of Laboratory Animals and complied with protocols approved by the Institutional Animal Care and Use Committee at the Houston Methodist Research Institute (AUP‐0219‐0013). Briefly, C57BL/6 female mice were divided into 3 groups (*n* = 5 mice per group) and injected intraperitoneally with 5 × 10^6^, 1 × 10^7^, or 1.5 × 10^7^ ID8‐Luc/GFP cells in 200 μL of PBS. Cells were injected into the lower right quadrant of the abdomen. Mice weights (g) were recorded daily following ID8‐Luc/GFP cell injection and plotted as fold change. Representative macroscopic images of tumors and ascites development were taken with a smartphone camera.

### 2.4 Bioluminescence, imaging, and tumor localization within the abdominal cavity

To track tumor growth, luciferase luminescence was detected using a Xenogen IVIS Spectrum imaging system (PerkinElmer) as previously described.^25^ Briefly, 200 μL of 15 mg/mL D‐luciferin was injected into the mice abdomen and the bioluminescent signal evaluated after 10 min to obtain the peak photon emission per second. The signal was quantified using the Living Image software (PerkinElmer) and the total photon flux emission (photons/second) in the regions of interest (ROI) recorded, starting at day 8 after tumor cell injection. Images were normalized using the Living Image software (PerkinElmer) with a minimum and maximum radiance of 1.7 × 10^4^ and 9.7 × 10^4^ photons/s, respectively.

### 2.5 Hematoxylin and eosin (H&E) staining and immunohistochemistry (IHC)

Sixty‐three days after ID8‐Luc/GFP cell injection, mice had a strong tumor signal intensity by IVIS. Hence, mice were sacrificed and the peritoneal membrane, abdominal tumors, and liver were sampled, fixed in 4% paraformaldehyde solution overnight, and embedded in paraffin. Paraffin embedded tissues were subsequently sectioned at a thickness of 5 μm and hematoxylin and eosin (H&E) staining performed to enable general inspection of the tissues. The 5 μm thick sections were also used for immunohistochemical staining. Sections were incubated with primary anti‐CD3 (rabbit, Dako), anti‐MHC‐II (rat, eBioscience), or anti‐F4/80 (rat, BioRad) for 1 h at room temperature (RT) in a moist chamber. Sections were imaged with a EVOS® FL Auto Imaging System (Life Technologies).

### 2.6 Ascites extraction and mass cytometry by time of flight (CyTOF) analysis

Seventy days after tumor cell injection with 1 × 10^7^ ID8‐Luc/GFP cells, mice started developing ascitic fluid. Ascites onset was detected by abdomen palpation, by eye and by weight increase. After reaching a weight of 30 g, three mice were sacrificed, and the ascetic fluid collected by syringe suction following abdominal incision. Ascitic fluid was centrifuged, and red blood cells lysed by incubation in Ammonium‐Chloride‐Potassium (ACK) lysing buffer (ThermoFisher) for 10 min at RT. Immune cell enrichment was achieved using Percoll gradient centrifugation. Briefly, the cell pellet was resuspended in 85% Percoll (GE Healthcare), then carefully layered onto 50% Percoll and centrifuged at 620 × *g* without the brake for 30 min at 4°C. After centrifugation, three layers of cells were present. The middle layer, consisting of the immune cells of interest, was recovered and used for mass cytometry staining. Cell viability was determined by incubation with 25 μM cisplatin for 5 min. At these conditions, cisplatin preferentially reacts with proteins in dead cells and it widely established as a viability reagent for mass cytometry.^26^ After washing in Maxpar® Cell Staining Buffer (Fluidigm), cells were resuspended in 40 μL surface‐staining antibody (Ab) mix and incubated at RT for 30 min. Antibodies were purchased from Biolegend (except for Arginase‐1 and NOS2, which were purchased from eBioscience) and conjugated to the metals using the Maxpar® X8 Multimetal Labeling Kit (Fluidigm). Selected Ab are shown in Table 1. Cells were then washed 2× and fixed with 100 μL of Fix/Perm buffer (eBioScience) for 10 min, followed by the addition of 200 μL Perm buffer (eBioScience) for 10 min. The intracellular staining was performed by diluting cells in 50 μL Ab mix and incubating them at RT for 60 min. After the washing steps, the cell ID DNA intercalator (500 μM, Fluidigm) was added to cells in a 1:1000 dilution for 30 min at RT. Cells were then washed, counted, and filtered through blue‐capped tubes (35 μm) before resuspension in 50 μl deionized water and the addition of 50 μl of EQ‐beads (eBioScience). Samples were acquired by Helios CyTOF machine. A total of 100 000 events were recorded for each sample, and subsequently analyzed on Cytobank. Immune cell populations were identified by manual gating. The intensity of the signal in the viSNE plots obtained was divided into three main groups. The following thresholds were used for categorization of the immune cell subtypes and applied to each specific viSNE plot: if the majority of the region was in the high range of expression (the red colored area, with numerical values varying according to the analyzed marker), the marker was considered highly expressed (++); if the region was in the middle range of expression (color‐coded azure to orange) then the marker was considered to be moderately lowly expressed (+). Finally, if the area exhibited mostly lower expression (indicated by blue and dark blue colors), markers were considered not expressed (−).

**TABLE 1:** Panel of the antibodies selected for mass cytometry, CyTOF. List of the 33 antibodies used, and their metal conjugation

Target	Metal tag
CD45	Pr141
MHC II	Nd142
CD11b	Nd143
Ly6C	Nd144
Ly6G	Nd145
F4/80	Nd146
CD11c	Sm147
CD38	Nd148
Arg‐1	Sm149
SiglecF	Nd150
CD206	Eu151
CD62L	Sm152
CD103	Eu153
iNOS	Sm154
PD‐L1	Gd155
TNFa	Gd156
CD64	Gd158
TCRgd	Tb159
Foxp3	Gd160
RORgt	Dy161
CD8α	Dy162
Tbet	Dy163
CD25	Dy164
IFN‐γ	Ho165
CD44	Er166
CD86	Er167
CD80	Er168
PD‐1	Tm169
B220	Er170
NK1.1	Yb171
CD19	Yb173
CD4	Yb174
TCR β	Lu175

### 2.7 Statistical analysis

Statistical analysis was performed by ANOVA for all experiments that required it. More specifically, a two‐way ANOVA with post hoc Dunnett comparisons to week 1 or Day 0 was employed for tumor signal and tumor weight analyses, respectively. Data with a *P* < 0.05 were considered significant (∗*P* < 0.05, ∗∗*P* < 0.01, ∗∗∗*P* < 0.001). All results were obtained from independent experiments and expressed as the mean ± standard deviation (SD).

## 3 RESULTS

### 3.1 Tumor model optimization

**FIGURE 1:**
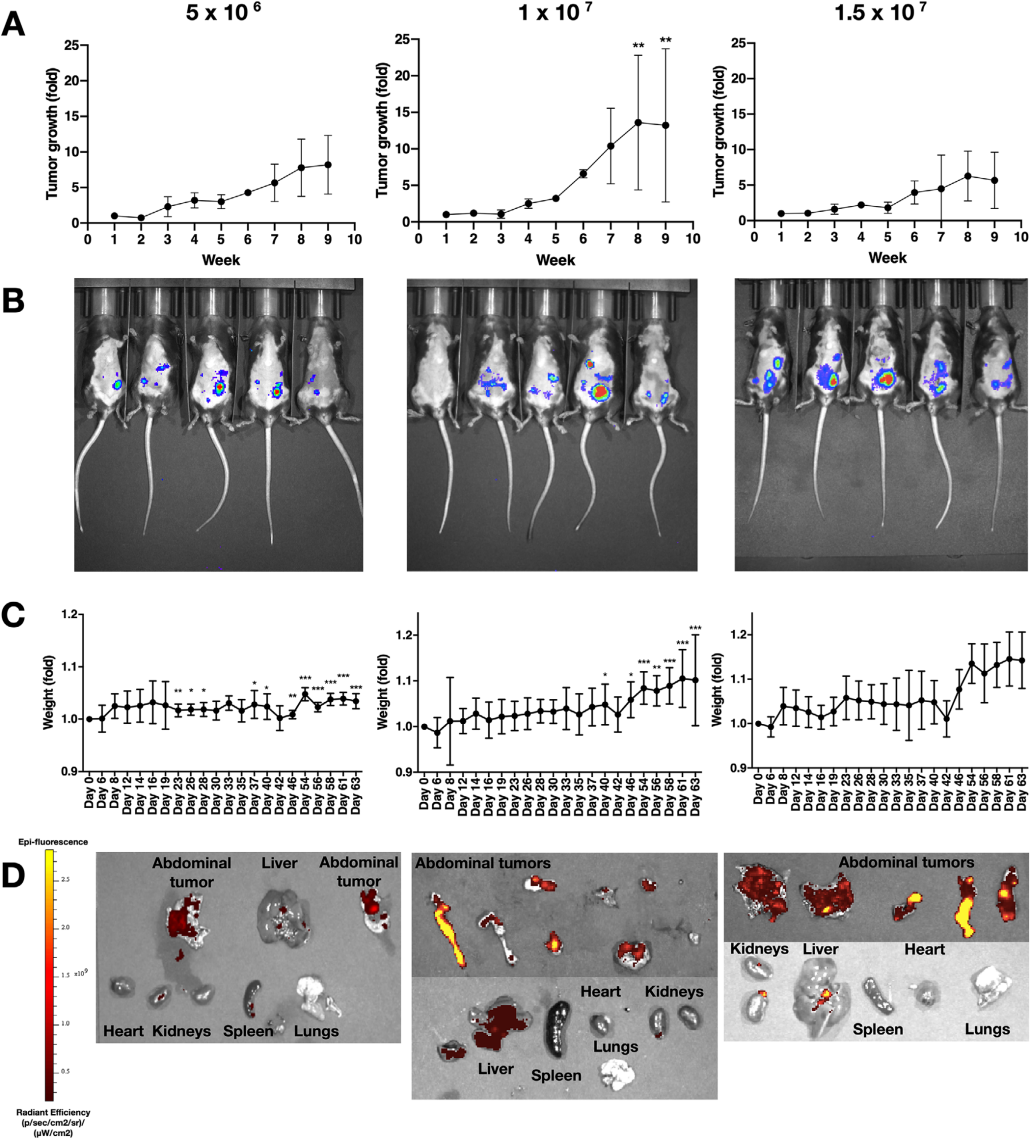
Tumor model generation and optimization. (A) Tumor growth signal quantification over a 9‐week period by IVIS following injection of 5 × 10^6^, 1 × 10^7^, or 1.5 × 10^7^ cells (*n* = 5). (B) Representative IVIS images of each tumor group taken after 6 weeks from tumor cells injection. (C) Mice weights (grams), expressed as fold change, during the 9‐week experimental period (*n* = 5). (D) IVIS images of organs extracted from the mice abdomen 9 weeks after injection with 5 × 10^6^, 1 × 10 ^7^, or 1.5 × 10^7^ ID8‐Luc/GFP cells. Extracted organs include liver, spleen, kidneys, lungs, heart, peritoneal membranes, tumor nodules. Epi‐fluorescent signals reflects the presence of ID8‐Luc/GFP cells. Data are expressed as mean (SD) from 5 independent experiments. Data were analyzed by ANOVA and Dunnett's pairwise multiple comparison test; values differ from week 1 (A) or day 0 (C), *P < 0.05, **P < 0.01, ***P < 0.001

After ID8‐Luc/GFP cells (5 × 10^6^, 1 × 10^7^ or 1.5 × 10^7^) were intraperitoneally injected, tumor growth was assessed over a 9‐week period. After 9 weeks, animals injected with 1 × 10^7^ cells showed a 13.6 ± 9 fold increase in tumor growth, based on fluorescence signal, compared to week 1 (*P* < 0.001), as quantified by IVIS (Figure 1A). Mice injected with 5 × 10^6^ or 1.5 × 10^7^ cells showed a 8.2 ± 4 fold and 5.6 ± 43.9 fold increase in tumor growth, respectively, again based on fluorescence signal, compared to week 1 (*P* < 0.001). No differences in tumor growth were noted between the three treatment groups, although all three groups showed increased tumor size over time. Representative pictures of the signal produced by the tumor within the abdomen are shown in Figure 1B. Figure 1C shows the fold variation of mice weights over the 9‐week period, indicating a correlation between tumor growth and mouse weight, which was particularly evident following injection of 1 × 10^7^ or 1.5 × 10^7^ cells. Statistical difference was seen within groups at different time points when compared to the day of injection (*P* < 0.001). This difference became more marked after day 23 days in 5 × 10^6^ group and after 37 days in 1 × 10^7^ group. No statistically significant intragroup differences were recorded in 1.5 × 10^7^ group. Sixty‐three days after tumor cell injection, mice were sacrificed and organs extracted to better localize the tumors within the abdomen. ID8‐Luc/GFP cells were identified in several abdominal organs including liver, kidneys, and spleen, and multiple tumor nodules/formations were randomly distributed around the abdominal cavity within the peritoneum (Figure 1D).

### 3.2 Histological analysis of liver and tumor nodules

**FIGURE 2:**
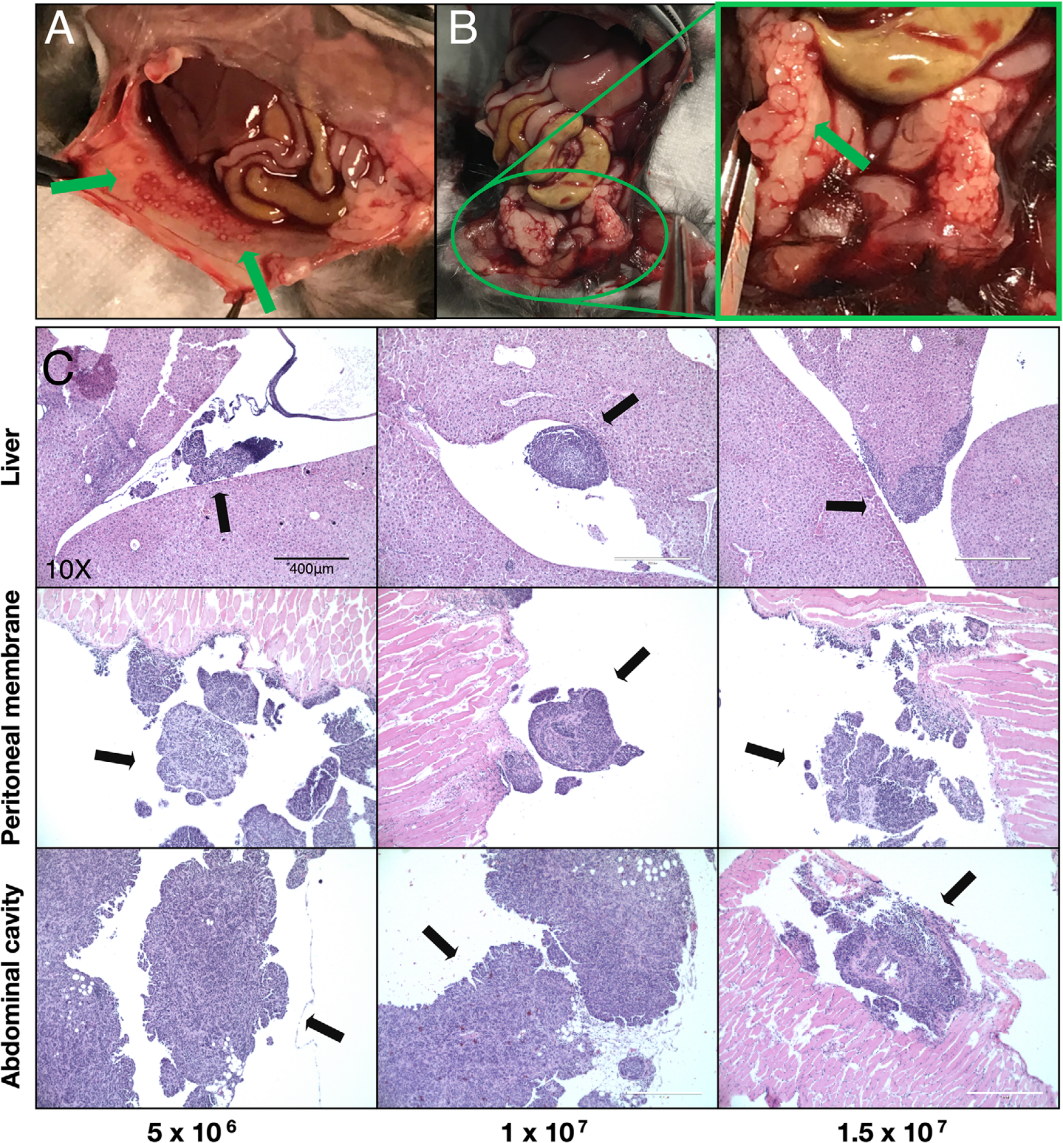
Histological assessment and localization of tumor nodules within the peritoneal cavity. (A) Representative images of tumor growth on the peritoneal membrane (green arrows). (B) Representative images of tumor growth within the lower abdominal cavity, indicated by the green arrow in the inset image. (C) H&E staining of tumor nodules found in the liver, peritoneal membrane, and abdominal cavity (black arrows). Magnification: 10×, scale bar: 400 μm

Tumors, extracted at day 63 after intraperitoneal injection, were processed and stained with H&E. Tumor growth occurred in two main areas; firstly the inner surface of the peritoneal membrane, as can be inferred from nodules visible in mice from the 1 × 10^7^ group (Figure 2A), and second, the abdomen, where multiple tumor masses were found in multiple organs of the lower abdomen (Figure 2B). H&E staining of the liver, peritoneal membrane, and tumor masses within the abdominal cavity shows the presence of tumor growth within all three treatment groups and illustrates the extent of tumor infiltration throughout the peritoneal cavity (Figure 2C).

### 3.3 Immunohistochemistry of immune infiltrates within tumors

**FIGURE 3:**
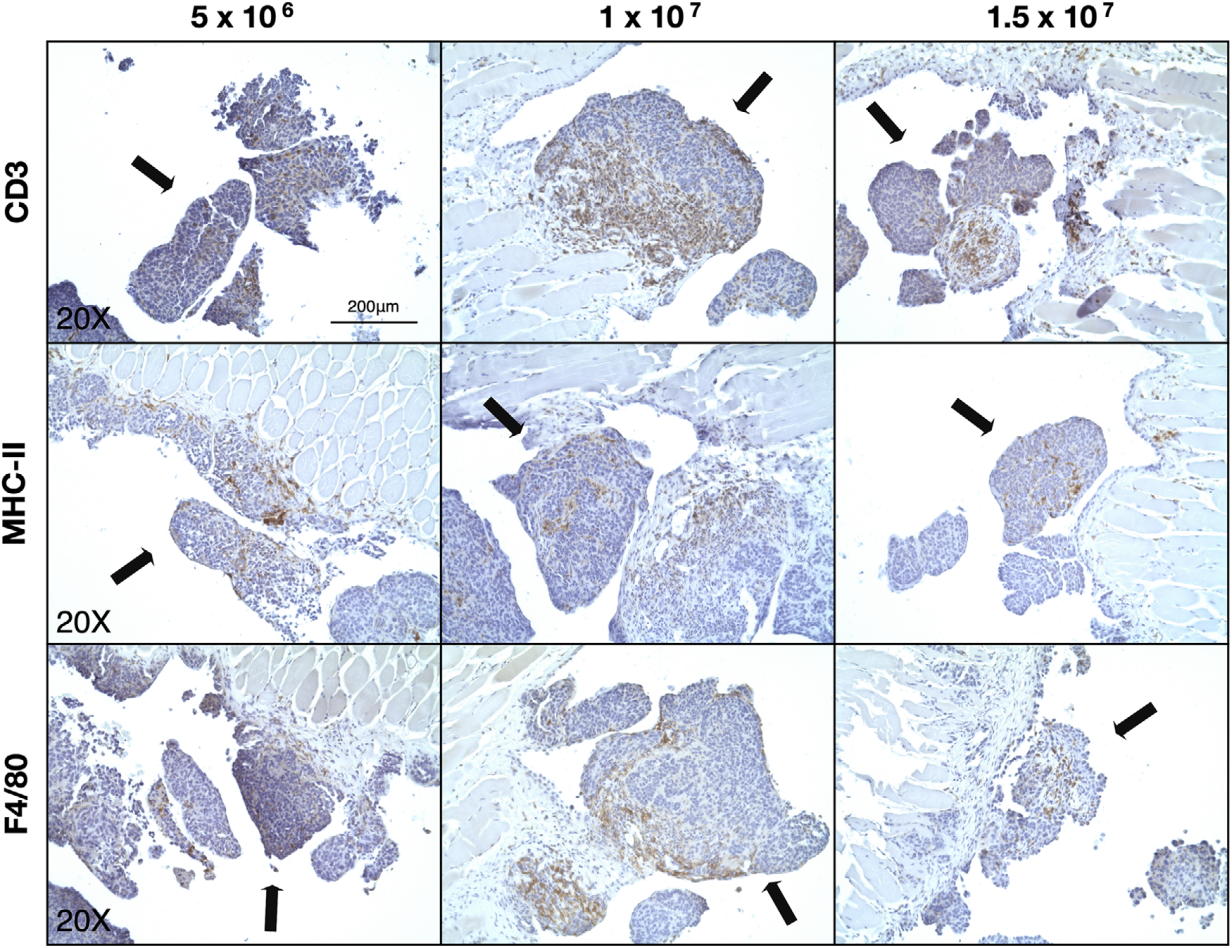
Identification of immune cell infiltration within tumors of the peritoneal membrane. Representative IHC images of each of the experimental groups showing the presence of CD3+, MHC‐II+, and F4/80+ cells (T cells, APC, and macrophages, respectively) within tumors on the peritoneal membrane. Black arrows indicate the tumor masses. Magnification: 20×, scale bar: 200 μm

Tissue sections were also analyzed by immunohistochemistry (IHC) to identify immune cell infiltration within tumors found on the peritoneal membrane. The presence of cell surface markers for T cells, antigen presenting cells (APC), and macrophages (CD3, MHC‐II, and F4/80, respectively) was investigated in mice from all treatment groups (Figure 3). Significant immune cell infiltration was apparent in tumors present in all the treatment groups. Of note, there were high numbers of T cells and macrophages, indicating an active immune environment.

**FIGURE 4:**
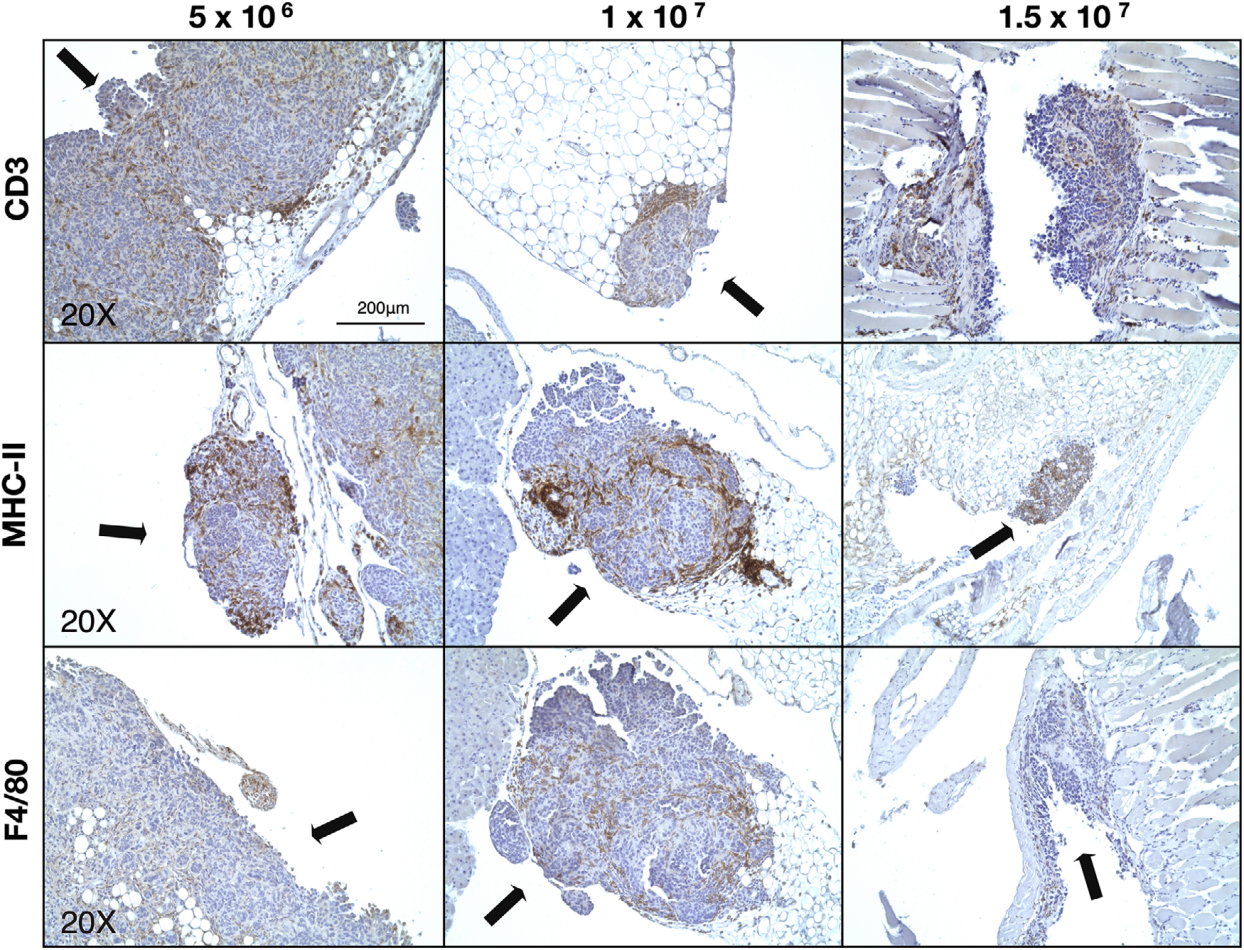
Identification of immune cell infiltrates within abdominal cavity tumors. Representative IHC images of CD3+, MHC‐II+, F4/80+ cells (T cells, APC, and macrophages, respectively) within tumor nodules (indicated by black arrows) found within the abdominal cavity. Magnification: 20×, scale bar: 200 μm

A similar trend of immune cells recruitment to the tumor masses was observed in the cancerous fragments extracted from different areas of the abdominal cavity. Figure 4 shows a strong presence of immune infiltrates despite the varied histological landscapes of the tissues examined.

### 3.4 Immune characterization of ascites through mass cytometry (CyTOF)

**FIGURE 5:**
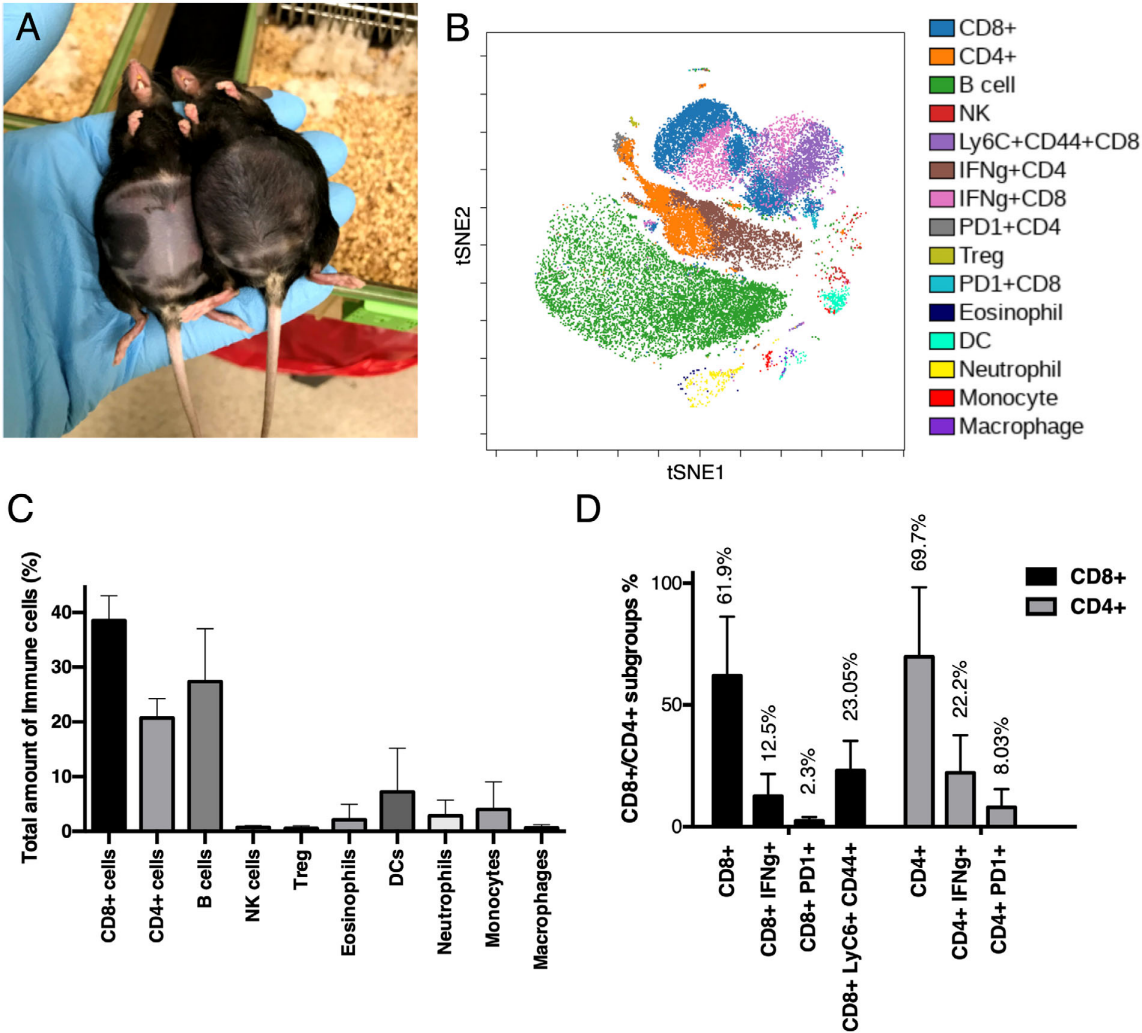
Characterization of immune cell populations in ascites. (A) Representative image of ascites formation in tumor‐bearing C57BL/6 mice 70 days after tumor cell injection. (B) visNE plot obtained by mass cytometry depicting the most represented immune cell populations in ascites collected from tumor‐bearing mice (*n* = 3). (C) Immune cell populations identified from the mass cytometry analysis as a percentage of the total cells. (D) Percentages of CD4+ and CD8+ T‐cell subtypes, which are mainly CD4+/CD8+/IFNγ+, CD4+/CD8+/PD1+, or CD8+/LyC6+/CD44+. Data presented are mean (SD)

To obtain a better understanding of the immune landscape of our metastatic OC model, immune cell populations within the ascites of tumor‐bearing mice were analyzed by mass cytometry. Seventy days after tumor cell injection, ascitic fluid formation resulted in a swollen abdomen that was apparent and palpable (Figure 5A). Differential expression analysis of specific immune cell surface markers (Table 1) present on CD45+ cells within ascitic fluid was performed through mass cytometry (CyTOF) analysis. Results from this analysis are plotted onto a viSNE graph (Figure 5B) that plots CD45+ cells on a two‐dimensional map and identifies individual cells by their expression of the specific immune cell markers chosen (for gating strategy, see Figure S1). From these data, immune cell population percentages and numbers can be derived (Figure 5C) enabling a comprehensive analysis of the ascitic fluid immune cell population. Within the present study, the most abundant cell populations in the ascitic fluid of tumor‐bearing mice were B cells (27.3.6% ± 9.6%), CD8+ T cells (38.5% ± 4.5%), and CD4+ T cells (20.7% ± 3.5%), with myeloid immune cells, including monocytes, macrophages, DCs, eosinophils, and neutrophils accounting for the remaining 15% of the total cell population.

Further characterization revealed the presence of specific subpopulations among CD8+ and CD4+ T cells (Figure 5D). Specifically, based on their expression of specific markers we identified the presence of (i) memory CD8+ T cells (Ly6C+/CD44+, 23.1% ± 12.1%), (ii) T helper cells (IFNγ+/CD4+, 22.2% ± 15.3%), and (iii) cytotoxic T lymphocytes (CTL, IFNγ+/CD8+, 12.5% ± 9.05%). In addition, low expression of PD1 in 8.03% ± 7.4% of CD4+ T cells and 2.3% ± 1.5% of CD8+ T cells was noted indicating a low level of T‐cell exhaustion. The percentages of remaining CD8+ and CD4+ T cells with no identified subpopulations were 61.9% ± 24.3% and 69.7% ± 28.5%, respectively. The CD8+/CD4+ T‐cell ratio was 1.65, indicating a prevalence of CD8+ T cells, while the relative percentages of T helper and CTL cells (4.6% ± 3.1% and 4.85% ± 3.4%, respectively) were similar.

Table 2 shows the list of all markers identified in each immune cell population and differentiates them based on the level of expression (see Section 2.6). For instance, the B‐cell group is characterized by the high expression of the markers CD38, B220, MHC‐II, CD19, and CD80. In addition, CD25, PD‐L1, and PD‐1 are found to be expressed by both B cells and CD4+ and CD8+ cells at high and low expression levels, respectively. CD4+ and CD8+ cells share high expression levels of TCRβ and IFN‐γ (in addition to the cell specific markers CD4 and CD8α, respectively), with Ly6C, Tbet, CD25, and CD103 being specifically expressed by the CD8+ population. Treg cells express high levels of Foxp3, CD25, CD4, and CD44 markers among others, with CD44 being also present in eosinophils, NK cells, monocytes, macrophages, DCs, and neutrophils. Macrophages show high expression of F480 and share with monocytes the moderate to low expression of CD11b, TCRgt, CD64, and CD80. Similarly, the presence of CD11b, Ly6C, and Ly6G is observed in both, eosinophils, and neutrophils.

**TABLE 2:** Expression level of markers identified in each immune population found in the ascitic fluid

	B cells	CD8+ cells	CD4+ cells	T regulatory	NK cells	Monocytes	Neutrophils	Eosinophils	Macrophages	Dendritic cells
CD38	++	−	−	+	−	+	+	−	+	+
B220	++	−	−	−	−	−	−	−	−	−
CD25	+	+	−	++	−	−	−	−	−	−
TCRb	−	++	++	++	−	−	−	−	−	−
IFN‐γ	+	++	++	−	−	−	−	−	−	+
MHC‐II	++	−	−	+	−	−	−	−	+	+
TNF‐α	+	−	−	−	−	−	−	−	−	−
PD‐L1	++	+	+	+	−	−	−	−	+	+
CD4	−	−	++	++	−	−	−	−	−	−
Tbet	−	+	−	−	+	−	−	−	−	++
CD19	++	−	−	−	−	−	−	−	−	−
CD80	++	−	−	−	−	+	+	+	+	−
PD‐1	+	+	+	+	−	−	−	−	−	−
CD8a	−	++	−	−	−	−	−	−	−	−
CD103	−	+	−	+	−	−	−	−	−	−
CD86	+	−	−	+	−	+	+	+	−	−
F480	−	−	−	−	−	+	+	+	++	−
CD45	−	−	−	−	−	−	−	−	−	−
RORgt	−	+	−	−	−	−	−	+	−	−
Ly6C	−	+	−	−	−	++	++	++	−	−
CD11b	+	−	−	−	−	+	++	++	+	++
CD64	−	−	−	−	−	+	−	−	+	−
CD44	++	++	+	++	++	++	++	++	++	++
TCRgt	−	−	−	−	−	+	+	+	+	−
Arg‐1	−	−	−	−	−	−	−	−	−	−
NK 1.1	−	−	−	−	++	−	−	−	−	−
CD11c	−	−	−	−	+	−	−	−	−	++
Ly6G	−	−	−	−	−	−	++	++	−	−
SinglecF	−	−	−	−	−	−	−	++	−	−
Foxp3	−	−	−	++	−	−	−	−	−	−
iNOS	−	−	−	−	−	−	−	−	−	−
CD62L	−	−	−	−	−	−	−	−	−	−
CD206	−	−	−	−	−	−	−	−	−	−

The immune markers were assigned to each category of CD45+ cells according to their expression levels in that specific subpopulation. Marker intensity thresholds used to discern between high and low marker expression are reported in Section 2.6 of methods. ++: high expression, +: intermediate expression, –: lack of expression. iNOS, CD62L, and CD206 were not expressed on any cell population within the ascites.

**FIGURE 6:**
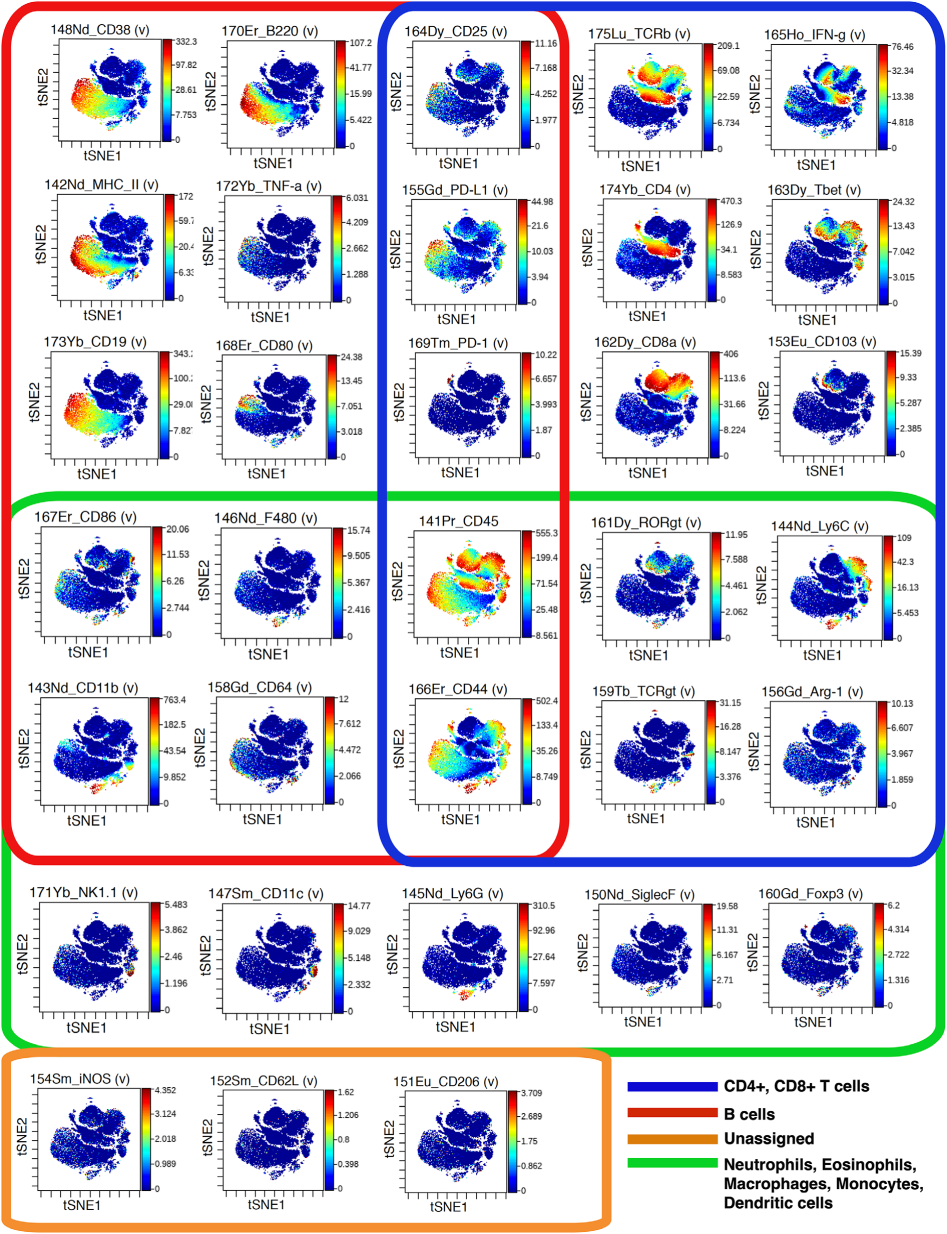
viSNE plots for 33 immune markers. From the CyTOF analysis of the 33 immune cell markers, three main immune cell populations were identified based on the coexpression of specific markers. B cells: CD38+/B220+/MHC‐II+/TNFα+/CD19+/CD80+. T cells (CD4+ and CD8+): CD25+/PD‐L1+/ PD‐1+ have been colocalized on both, CD4+ and CD8+ cells, which also express subgroup‐specific markers TCRβ, IFN‐γ, CD4, CD8α, Tbet, and CD103. The third subgroup is represented by neutrophils, eosinophils, macrophages, monocytes, and dendritic cells, which are specifically positive for NK1.1, CD11c, Ly6G, SiglecF, and FoxP3. The markers CD86, F480, CD11b, and CD64 are shared with the B cells subgroup, whereas RORgtm Ly6C, TCRgt, and Arg‐1 are shared with the CD4+/CD8+ cell subgroup

To complement the information provided in Table 2 and visualize the immune cell marker expression, data from the CyTOF experiments were arranged into subgroups according to marker expression and their association with specific cell types (Figure 6). Several immune markers were associated with more than one cell population; however, the three main cell types identified were: B cells; CD4+ and CD8+ T cells; neutrophils, eosinophils, macrophages, monocytes, and dendritic cells. The expression of CD62L, iNOS, and CD206 was not apparent on any cell population.

## 4 DISCUSSION

The treatment of advanced OC is challenging, especially considering the altered physical transport properties that create an immunosuppressive environment^27^, ^28^ and limit responsiveness to current immunotherapy strategies. Efforts to optimize animal models that comprehensively mimic cancer development in vivo and thus enable new therapeutic strategies to be tested are ongoing. In this context, the mouse ovarian surface epithelial cell line (ID8 cell line) is widely used to generate preclinical models of advanced OC. This is due to its capacity to closely reproduce the histopathological nuances that are characteristic of patients with advanced OC. These include tumor dissemination across the peritoneal cavity, a specific pattern of invasion and the formation of ascites, which further increases the metastatic process and hinders therapy effectiveness.^29^ In addition, ID8 cells have been found to express Pax8,^30^ a member of a transcription factor family that has also been linked to a role in OC development.^31^ Lastly, the immunological nature of ID8‐based OC models further increases their clinical relevance as they also allow for the testing of innovative immunotherapies with the potential to treat this malignancy. As such, ID8 cells have been widely used to test different hypotheses. For example, Wilson et al used the ID8 model to track nuclear factor‐kappa B (NF‐κB) signaling during cancer progression,^32^ while Zhang et al used ID8 cells stably expressing the vascular endothelial growth factor to demonstrate increased tumor‐progression rate and ascites formation.^33^ However, some doubts about the stability of this cell line have been raised, since the onset of ascites has been reported to alter the efficacy of the bioluminescent signaling associated to ID8‐Luc/GFP cells.^34^ Moreover, despite ID8 cells being considered the gold standard when generating advanced OC in immune competent mice,^35^ the scientific community is yet to provide robust protocols, nor a consensus on optimal cell concentrations and incubation times for tumor development. In particular, the literature reports a wide range of ID8 cell concentrations being peritoneally injected (between 1 × 10^6^ and 1 × 10^7^),^32^, ^36^, ^37^, ^38^, ^39^ and different incubation times required to develop a noticeable tumor and ascites in immunocompetent mice.^34^, ^39^

In this work, we tested three concentrations of ID8‐Luc/GFP cells (5 × 10^6^, 1 × 10^7^, or 1.5 × 10^7^ cells) for their capacity to develop an advanced tumor in immune‐competent mice after intraperitoneal injection, with the aim of identifying a robust, reproducible protocol for tumor development. The range of concentrations selected was based on the most remarkable results found in literature, that is, significant tumor and acsities development. Our results demonstrated significant tumor growth over a 9‐week period for each of the cell concentrations tested, suggesting the suitability of these cell concentrations to reliably create a tumor in vivo. Additionally, the onset of ascites was confirmed between 70 and 80 days after cell injection, which was associated with increased mouse weight providing an indication of advanced stage disease.

H&E staining confirmed the presence of distinct tumor nodules on the liver, scattered across the abdominal cavity and lining the peritoneal membrane. No differences in the number or size of nodules between the experimental groups was evident microscopically. Thus, further characterization to assess the immune environment within the tumor nodules was undertaken. In agreement with the existing literature on immune cells present within the OC TME,^40^ immunohistochemical analysis showed T cells and antigen presenting cells (macrophages and DCs) distributed throughout tumor nodules found on the surface of the peritoneal membrane and scattered within the abdomen. The presence of CD3+ tumor infiltrating lymphocytes (TILs) has been identified as an independent prognostic factor in patients with epithelial OC.^41^, ^42^, ^43^, ^44^, ^45^ In addition, antigen presenting cells such as tumor associated macrophages (TAMs) and DCs have significant roles in the TME. In particular, TAMs, the most represented cell population,^46^ have the potential to suppress or stimulate an anticancer response according to the effect the surrounding microenvironment exerts on them.^47^, ^48^

Focusing on the ascites, which is closely linked to an altered immune environment within the peritoneal cavity of advanced OC patients, further characterization was undertaken. To comprehensively analyze the immune landscape of the ascitic fluid collected from an ID8 ovarian cancer model, for the first time we exploited mass cytometry. The 33 immune cell markers analyzed allowed for the specific identification of distinct immune cell populations and their linkage to pivotal functions with respect to tumor progression. Compared to the review from Wertel et al mentioned above, our results showed similar overall percentages of CD8+ T cells, in contrast with the lower percentages of CD4+ T cells and B cells.^49^ The CD8+/CD4+ T‐cell ratio of 1.76 we identified was indicative of a higher overall presence of CD8+ T cells compared to CD4+ cells, which is associated with improved patient survival. This finding, together with the expression of IFNγ, suggests effective immune stimulation within the tumor and the development of cell‐mediated immunity. In contrast, Giuntoli et al demonstrated that a high CD4+/CD8+ T‐cell ratio is associated with poor outcome done in patients with ovarian, primary peritoneal or fallopian tube cancers, and that high concentrations of interleukins 6 (IL‐6) and 10 (IL‐10) can help establish an immunosuppressive climate that might lead to a decreased activation of ascites‐derived T cells.^50^

**FIGURE 7:**
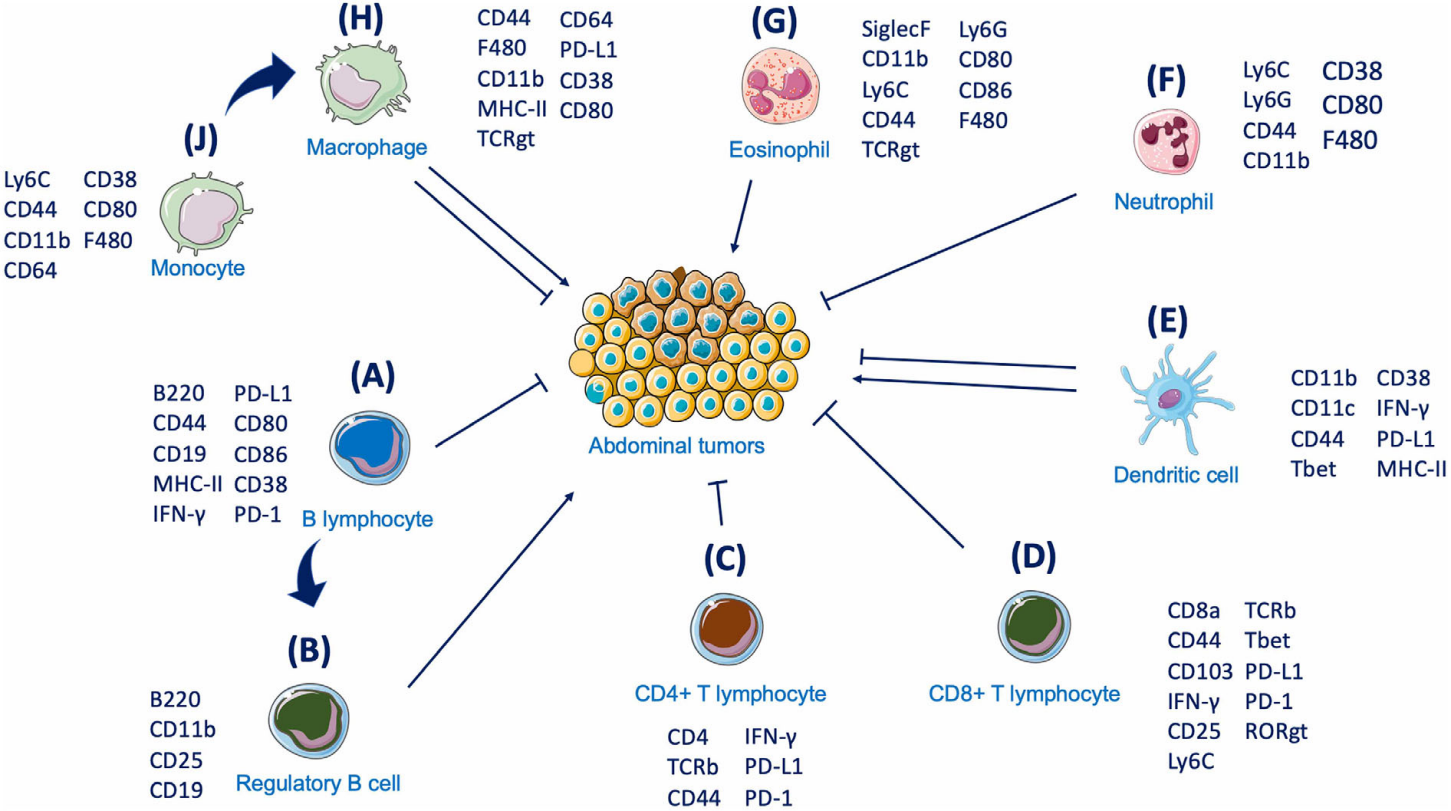
Schematic of ascites immune cells and their interactions with the tumor. The interactions between ascites immune cells and their potential effect on tumor cells. B220+/CD11b+/CD38+ B lymphocytes (A) and their CD25+/CD119+ regulatory B‐cell subgroup (B) exert antitumor and protumor activity, respectively. The role of CD4+ (C) and CD8+ (D) T cells in exerting cytotoxic activity towards the tumor is linked to the antigen presenting capacity of CD11b+/CD11c+/CD44+ DCs (E). Eosinophils (G) and neutrophils (F) are linked to protumoral and antitumoral properties, respectively. In parallel, monocytes and macrophages (H‐J) exert either a tumor promoting or suppressive effect according to the surrounding microenvironment

In Figure 7, we summarize the possible interactions occurring between the immune cell populations identified within the ascites and their effect on the metastatic tumors in situ, based on cell‐surface marker expression. The presence of CD45+B220+ B cells can be linked to both pro‐ and antitumor responses due to their phenotypical and functional variability, as confirmed by several other studies. Indeed, the B220+CD11b+MHC‐II+ B‐cell population (7A) can have a positive or neutral prognostic effect,^51^, ^52^ which can also be mediated by CD38 expression.^53^ In contrast to this, the presence of CD25+CD19+ B regulatory cells (7B) is correlated with suppressed T cells responses and poorer patient survival.^54^

Two additional markers, PD‐1 and PD‐L1, present on both CD4+ (7C) and CD8+ T (7D) cells, have a pivotal role in establishing efficient immunotherapeutic approaches, after it was demonstrated that their inhibition can stop cancer progression.^55^ Although clinical trials testing PD‐1 and PD‐L1 inhibitors have not yet yielded satisfactory results in OC as single treatment,^8^, ^9^ their use as combinatorial treatment still holds promise. The prognostic value of PD1+ TILs, when colocalized with PD‐L1 on cancer cells has been demonstrated supporting the PD‐1 inhibitory pathway as one mechanism they use to silence the immune system during OC progression.^56^ In particular, although tumors appear to be infiltrated by T cells at early stages, a progressive reduction in the frequency of CD8+ T cells and CD8:Treg ratio was noticed at more advanced advanced stages.^57^ Our findings have also attributed the majority of PD‐L1 expression to macrophages (**7H**) and, together with the presence of cytolytic and regulatory TIL subsets, link directly to survival potential.^58^ Macrophages, identified through the coexpression of F4/80 and CD64, were also positive for MHC‐II, CD44, and CD80, with the latter marker suggesting an M1 phenotype, which has been linked to increased inflammatory status^47^ and is specifically correlated with a longer overall survival (OS) and progression‐free survival (PFS) in serous OC patients.^59^

Neutrophils (**7F**), identified by the presence of the markers Ly6C and Ly6G (similar to other myeloid derived populations such as eosinophils (**7G**) and monocytes/macrophages), were also present.^60^ Neutrophils have been connected to antitumor‐promoting activity in OC. Indeed, neutrophils isolated from the ascites of a KRAS‐ID8‐induced mouse model showed KRAS‐dependent CD8+ T‐cell activation through increased recruitment of costimulatory molecules. On the contrary, neutrophil depletion (through administration of an anti‐Ly6G monoclonal antibody) led to marked tumor progression.^37^ More recently, however, Ly6G‐positive neutrophils have been reported to promote a microenviroment that is conducive of metastases spreading and accumulation at specific sites.^61^

Dendritic cells, identified by the expression of CD11b+, CD11c+, CD44+, and MHC‐II (**7E**), are paramount players in the activation of effective T‐cell responses through their antigen‐presenting activity. Indeed, CD44 was found to be pivotal in the formation of tight junctions between mature DCs and T cells and to play a role in T‐cell activation as a consequence.^62^ However, DCs can undergo tumor‐mediated immunosupressive processes, such as the blockage of their activity through the tumor‐induced upregulation of the unfolded protein response (UPR), as showed by Cubillos‐Ruiz et al.^14^ Moreover, Krempski et al also found that tumor infiltrating, PD‐1+/PD‐L1+ DCs within the ascites respond poorly to danger signal, suppress T‐cell activity and decrease T‐cell infiltration within the tumor masses.^63^

The immune cells identified within the ascites produced in this model of HGSOC are linked to both pro‐ and antitumoral activity, indicating that this model represents a balanced immune response to the tumor, or that the immunosuppressive effect of the tumor is yet to take hold. For instance, while expression of the integrin, CD103, and transcription factor, Tbet, associated with CD8+ T cells might indicate a better prognosis,^64^, ^65^, ^66^ the high expression of Ly6C on monocytes is a strong indicator of a TAM phenotype with strong immunosuppressive potential and poor a prognosis.^67^, ^68^

More generally, the presence of immune‐active components within the tumor nodules and the ascites raises the question of why the therapeutic potential of immunotherapies is still limited in OC settings. In this case, additional factors should be considered, including the so‐called tumor mutation burden (TMB). TMB results from the identification and quantification of driver genes mutations that are responsible for the production of neoantigens. The increasing presence of neoantigens has been associated to the activation of the antitumor immune response. For this reason, TBM plays an important role in the progression of a cancer with a high mutation load being associate to a better prognosis.^69^ A recent investigation calculating TMB in 397 patients with OC in the TCGA database revealed that resting immune cells (B cells, B cells, CD4+ T cells, Tregs, monocytes, mast cells, and neutrophils) likely infiltrate tumors with low TMB, whereas activated immune cells (CD4+ T cells, follicle‐assisted T cells, proinflammatory macrophages) infiltrate tumors with high TMB.^70^ In other cases, some cell‐based immunotherapies (such as CAR‐T) targeting a single tumor antigen often lose their efficacy as the result of mutations occurring in tumor cells, which impair specific antigen expression thus hindering the effect of the therapy.^71^ In addition, cell therapeutics often are subjected to the immunosuppressive environment they meet following administration, which limits their effectiveness in exerting an antitumor immune response.^28^, ^72^ The tryptophan catabolism offers another example relevant in this context, as the tryptophan‐catabolizing enzyme indoleamine 2,3‐dioxygenase (IDO) has been found to be hyperactive in OC and linked to the production of immunosuppressive catabolites and poor patient survival.^73^ In addition, the cancer‐induced acidic environment has been shown to have a role in tumor recurrence, metastasis, and prognosis of cancer patients (due to the high production of lactate).^74^ Furthermore, lactate can also support cancer cell immune evasion by inhibiting T‐cell activation^75^ and dendritic cell antigen presenting capacities.^76^

This work is the first to provide a multiparametric and comprehensive characterization of the immune cell landscape of the ascites collected from a preclinical model of advanced OC. Published literature reports fragmented information, as only single populations (such as CD4+ and CD8+ T cells) have been so far identified and described.^50^ A more complete description has been offered by Wertel et al who listed the percentages of the main cellular components found in the peritoneal fluid of advanced OC patients by merging the information collected from several different studies.^49^ More recently, the panorama of the ascites collected from HGSOC patients has been resolved by applying single cell‐RNA sequencing (scRNA‐seq).^77^ In this study, the authors provided a broad view of the different cell types in the ascites ecosystem, with particular focus on malignant versus nonmalignant cells (analyzing samples partially depleted of CD45+ immune cells).

The potential strength of the data we identified is therefore to demonstrate that mass cytometry provides a platform for the comprehensive analysis of the immune cell landscape within ascites, which would allow periodical analysis of cellular and molecular changes in patients with OC. In this regard, CyTOF holds the promise of complementing personalized therapeutic approaches, and potentially enabling real time tracking of the efficacy of immunotherapeutics. Compared to scRNA‐seq, CYTOF offers the advantage of a higher throughput for the evaluation of the TME in clinical samples, as it allows for a more accurate targeting of immune cell subsets through the use of >30 selected antigen markers. Moreover, CyTOF “narrow and distinct”^78^, ^79^ data are generated from the analysis of over 250 000 cells, whereas transcriptome‐based platforms detect wider unbiased populations from several thousands of cells.^80^, ^81^

## 5 CONCLUSIONS

In this work, we provide, for the first time, a comprehensive characterization of the immune landscape of the ascites collected from tumor‐bearing mice, unveiling its potential for clinical implementation. The continuous analysis of interactions between immune cells in a cancerous environment would significantly increase the number of therapeutic options for the treatment of this malignancy and offer a significant alternative for the evaluation of ongoing therapies. Data presented in this study prove mass cytometry as a promising tool to facilitate this process, with the potential to identify personalized therapeutic targets and establish improved immunotherapy strategies. In addition, the application of CyTOF on more complex and genetically modified mice models, as well as on patients’ derived samples, will also unveil new insights into disease heterogeneity, pathology, and drug resistance, and will expand our understanding of HGSOC.

## COMPETING INTEREST

The authors declare that they have no competing interests.

## ETHICS APPROVAL AND CONSENT TO PARTICIPATE

All animal studies were carried out in accordance with guidelines determined by the Animal Welfare Act and the Guide for the Care and Use of Laboratory Animals and complied with protocols approved by the Institutional Animal Care and Use Committee at the Houston Methodist Research Institute (AUP‐0219‐0013).

## AUTHORS' CONTRIBUTIONS

Conceptualization and methodology: SP, BC; Formal analysis and data curation: SP, SL, FI, LM, OSV, GDH, RSC and BC; Validation and investigation: SP, SL, LM and BC; Original draft preparation and Writing: SP, LM, GDH and BC; Review and editing: SP, GDH, DG, RSC and BC; Approval of final manuscript: all authors read and approved the final manuscript.

## AVAILABILITY OF DATA AND MATERIAL

All data relevant to the study are included in the article or uploaded as supplementary information.

## Supporting information

Supporting Information
