# Supplementary material for: Assessment of the immune landscapes of advanced ovarian cancer in an optimized in vivo model
Source: Clin Transl Med. 2021 Oct 12;11(10):e551. doi: 10.1002/ctm2.551 (PMC8506632; doi:10.1002/ctm2.551)
Supplement: Supplementary file 1 — Supporting Information [file CTM2-11-e551-s001.pdf]

Supplementary material

Population	Event Count	% of Total	% of Parent
Ungated	31271		
Cell	31271	100%	100% of Ungated
Singlet	31271	100%	100% of Cell
live	31271	100%	100% of Singlet
CD45+	31271	100%	100% of live
CD4+	6191	19.8%	19.8% of CD45+
IFNg+CD4	3468	11.09%	56.02% of CD4+
PD1+CD4	128	0.41%	2.07% of CD4+
Treg	73	0.23%	1.18% of CD4+
CD8+	7634	24.41%	24.41% of CD45+
IFNg+CD8	2639	8.44%	34.57% of CD8+
Ly6C+CD44+CD8	2253	7.2%	29.51% of CD8+
PD1+CD8	148	0.47%	1.94% of CD8+
Non CD4/CD8	16735	53.52%	53.52% of CD45+
B cell	13977	44.7%	83.52% of Non CD4/CD8
CD11b-	1602	5.12%	9.57% of Non CD4/CD8
NK	167	0.53%	10.42% of CD11b-
CD11b+	619	1.98%	3.7% of Non CD4/CD8
Eosinophil	18	0.06%	2.91% of CD11b+
SiglecF-CD11b+	596	1.91%	96.28% of CD11b+
DC	240	0.77%	40.27% of SiglecF-CD11b+
gateout DC	345	1.1%	57.89% of SiglecF-CD11b+
Neutrophil	124	0.4%	35.94% of gateout DC
Other Myeloid	217	0.69%	62.9% of gateout DC
Macrophage	28	0.09%	12.9% of Other Myeloid
Monocyte	104	0.33%	47.93% of Other Myeloid

Supplementary Figure 1. Gating strategy for Cytof analysis. Cells identified during the Cytof analysis were gated to identify specific immune cell populations according to their most representative markers. Cells that were identified as singlets and that were alive were first gated, followed by their positiveness to the immune-cell-wide marker CD45. Positiveness to CD4, CD8 was then used to gate all the immune subpopulations.
